# Perception of service users and their caregivers on primary care-based mental health services: a qualitative study in Nepal

**DOI:** 10.1186/s12875-020-01266-y

**Published:** 2020-09-28

**Authors:** N. P. Luitel, M. J. D. Jordans, P. Subba, I. H. Komproe

**Affiliations:** 1 Research Department, Transcultural Psychosocial Organization (TPO) Nepal, Kathmandu, Nepal; 2 Centre for Global Mental Health, Health Service and Population Research Department, Institute of Psychiatry, Psychology and Neuroscience, King’s College London, London, UK; 3 Faculty of Social and Behavioural Sciences; Department of Anthropology, University of Amsterdam, Armsterdam, the Netherlands; 4 Faculty of Social and Behavioural Sciences, Utrecht University, Utrecht, the Netherlands

**Keywords:** Mental health, Primary care, Integration, Service users, Perceptions, Nepal

## Abstract

**Background:**

Integration of mental health services into primary health care systems has been advocated as a strategy to minimize the tremendous mental health treatment gap, particularly in low- and middle-income countries. Barriers to integration of mental health into primary health care have been widely documented; however, very little is known about the perception of service users and their caregivers on primary care-based mental health services. This study assessed service users’ and caregivers’ perceptions of mental health services provided by trained primary health care workers in Nepal.

**Methods:**

A qualitative study was conducted among people with depression, psychosis, alcohol use disorder and epilepsy, and their caregivers in Chitwan, a district in southern Nepal. Semi-structured interviews were conducted with 43 service users and 38 caregivers to assess their perceptions about the accessibility of the services, types of services they received, skills and competencies of health care providers, satisfaction and barriers to receiving services.

**Results:**

Overall, both service users and caregivers were satisfied with the mental health services provided by primary health care providers. They also perceived health workers to be competent and skillful because the services they received were effective in reducing their mental health problems. Both psychological and pharmacological services were made available free of cost, however, they considered psychological services more effective than pharmacological treatment. Major challenges and difficulties accessing services were associated with frequent transfer of trained health workers, non-availability of the same health care provider at follow-ups, frequent stock-out of medicines or non-availability of required medicines, lack of a confidential space for consultation in health facilities, and stigmatizing and negative behavior of some health workers.

**Conclusion:**

The results demonstrated that both service users and caregivers perceived primary care-based mental health services to be accessible, acceptable and effective. The key recommendations emerging from this study for improving mental health services in primary care include the provision of a separate cadre of psychosocial workers to provide psychological interventions, developing quick and efficient mechanisms for the procurement and supply of psychotropic medicines, establishing a confidential place within health facilities for consultation, and further training of health workers to reduce stigma.

## Background

Mental, neurological and substance abuse (MNS) disorders are one of the leading causes of disability, contributing 10.4% of the global burden of disease in terms of disability adjusted life years (DALYs) [1]. A significant gap has also been reported between the number of people who need mental health care and those actually receiving services. Recent studies revealed that 86.3% of people with anxiety, mood or substance disorders [2] and 87% of people with alcohol abuse and dependence [3] had not received any treatment in the preceding 12 months. Among those who received treatment, 1 out of 27 people with depressive disorder and 1 out of 10 people with anxiety disorder received minimally adequate treatment [4, 5]. Even though various factors are considered to impede mental health care utilization, lack of mental health services or inability to afford the treatment cost are the most commonly reported barriers in low- and middle-income countries (LMICs) [6]. In recent years a great deal of evidence has been generated indicating that mental health services can be delivered effectively by trained community and primary health care workers through a task-sharing approach [7–9], and this approach has also been widely advocated as a strategy to reduce the mental health treatment gap, particularly in LMICs, where mental health specialists are limited and mental health resources are unequally distributed.

The World Health Organization (WHO) developed its Mental Health Gap Action Program (mhGAP) in 2008 [10] and mhGAP Intervention Guide (mhGAP-IG) in 2010 [11] in order to facilitate the integration of mental health services into primary care. The mhGAP intervention guide has now been used in more than 90 countries worldwide [12]. The PRogramme for Improving Mental health carE (PRIME), a research program consortium which aims to generate new evidence on the implementation and scaling-up of treatment programs for priority mental disorders, implemented and evaluated mhGAP-based district mental health care plans (MHCPs) in five LMICs [13, 14]. In Nepal, we trained prescribing health workers (medical officers, health assistants, and auxiliary health workers) on mhGAP-IG, and the nursing staffs (auxiliary nurse midwives and staff nurses) on basic psychosocial support. Trained health workers were supervised using combination of supervision methods - case conference, tele-supervision and direct observation. Prior studies have demonstrated a significant impact of the mental health services provided by trained primary health care workers on various indicators, including correct detection and initiation of minimally adequate treatment subsequent to diagnosis and individual level treatment outcomes [15]. Several studies have documented barriers to the implementation and scaling-up of mental health services in primary and community health care systems [16], however, these studies have focused on the perceptions of policy makers and service providers [17]. Very little is known about how service users and their caregivers perceive mental health services provided by trained primary health care workers, and the potential barriers they face to receiving these services. Therefore, in this study, we explored the experiences and perceptions of service users and their caregivers regarding primary care-based mental health services in Nepal.

## Methods

### Setting

The study was conducted in Chitwan, a district in Southern Nepal where the PRIME district MHCP was implemented, evaluated and scaled-up. The total population of Chitwan district is 579,984 (279,087 male and 300,897 female), with approximately 132,462 households. The literacy rate of Chitwan (78.9%) is higher than the national average literacy rate of 67% [18]. Chitwan is a diverse district in terms of caste/ethnicity, language, religion and geography. The major caste/ethnic groups in the district include Brahmin (28.6%), Chhetri (11.4%), Tharu (10.9%) and Tamang (8%) [18]. The study was conducted in 12 Village Development Committee (VDCs) covering a population of 117,000. Although mental health services in Chitwan district are available in the government district hospital and private medical colleges, there were no mental health services available in any of the 12 primary health facilities where the study was conducted.

### Participants and recruitment

The study was conducted with adults who were diagnosed with one of the priority mental disorders (i.e. depression, alcohol use disorder [AUD], psychosis, or epilepsy) and had initiated mhGAP-IG based treatment by trained primary health care worker, as well as their caregivers. All service users included in this study were participants in the PRIME cohort study, which evaluated the impact of PRIME MHCP in patient-level health outcomes [15]. A detailed overview of the cohort study can be found in Baron et al. [19]. Service users were selected purposively based on pre-defined criteria – i) 16 years or above ii) enrolled in the cohort study, iii) currently receiving primary care-based mental health services or recent dropouts from any of the 12 primary health care facilities. Caregivers of service users meeting abovementioned criterion were approached and enrolled in the study upon their consensus. All the invited service users and caregivers participated in the study.

### Sample size

The study was conducted with 43 service users [(depression (*n* = 12), psychosis (*n* = 8), AUD (*n* = 13) and epilepsy (*n* = 10)] and their caregivers (*N* = 38). Of the total 43 service users, 25 participants were regularly utilizing services whereas 18 participants had dropped out of services at the time of interviews.

### Data collection tools

Semi-structured interview guides were used for data collection. The interview guides were developed separately for service users and caregivers within the PRIME consortium, and then contextualized for Nepal. Interview guides were pilot tested with each category of the study participants before data collection commenced to ensure the questions were appropriate and understandable. Major themes included experiences of both service users and caregivers in receiving mental health services in primary care, types of services received, time spent with health workers, confidentiality, health workers’ attitudes toward their problems, health workers’ skills and competencies, satisfaction and barriers to receiving services. Data collection took place between September 2016 and May 2017. All interviews were conducted and recorded in Nepali language by an experienced team of researchers with university level education.

### Data management and analysis

The data were analyzed using a thematic analysis approach [20, 21]. The audio-recorded interviews were first transcribed in the original language (Nepali) by the interviewers immediately after the interviews. The transcriptions were then translated into English by professional translators. A systematic and iterative process was followed in data analysis. First, transcripts were read by two researchers to gain familiarity with the data and identify new themes and associated codes within each theme. Second, they coded 10% of the interviews separately and generated a coding framework using a priori coding framework based on the interview schedule, and the new themes identified during data familiarization for thematic analysis. Third, the coding framework was reviewed and finalized together with other team members involved in the development of topic guides and field data collection. Finally, QSR Nvivo 10 software was used for indexing and charting of the data based on the finalized coding framework [22].

## Results

### Accessibility of services

Although every primary health care workers at the 12 primary health care facilities were trained and all services (including psychological and pharmacological treatment) were made available free of cost, service users and caregivers expressed mixed opinions about the accessibility of the services. Service users and caregivers living in close proximity to the health facilities reported that the services were readily accessible whenever they needed. Other participants residing farther from health facilities reported difficulty accessing treatment because of the distance. Some service users hesitated visiting the health facilities often citing the expenses required for transportation. Both service users and their caregivers reported that it was more convenient to receive services from primary care than from private hospitals.

There were also many cases where the trained health workers contacted the caregiver or the service user periodically for their follow ups, and also to make sure patients are receiving medicines regularly. A caregiver of a participant with AUD shared her experience on how the primary health care workers supported her father through home-visit, *“they [primary health care workers] used to come to our home to give suggestions and convince my dad. They gave medication and used to call two or three times in a week for update, and ask how he is doing”.*

### Values and attitude of health workers

In general, participants reported having a good relationship with primary health care workers. They described the health workers’ behavior as ‘helpful’, ‘concerned’, ‘respectful’ and ‘supportive’, which was mainly because the health workers motivated them to use services and provided helpful suggestions (psycho-education and counseling). One of the patients with AUD reported:

*“I felt very good about their behavior. They provided information about my problem. They convinced me nicely about the importance of treatment for my [Drinking] problem. Later, I also realized that that the treatment was for me and not for them. I felt very good after talking to them. They treated me very well. I was given medicine as well. After taking the medicine, I did not even like the smell of alcohol.”* ***AUD Service user***

Contrastingly, another service user shared dissatisfaction when the health worker did not maintain confidentiality and spoke about his drinking habit with others in the community. Few service users and caregivers reported fearing that the health workers would scold them for not coming to services regularly or for not coming for follow-up.

Health workers not coming to the health facilities on time was another concern raised by both service users and their caregivers. They felt that some health workers were not serious about their responsibilities because they knew their job was secure regardless of how they performed. A few service users also reported that health care providers just provided medicine and sent them away without giving them much time to talk about their problems.

### Perceived skills and competency of health workers

Perspectives of service users and caregivers were similar regarding the skills and competency of the trained primary health care workers. The caregivers generally felt that the health workers were confident and competent to provide necessary services as they had seen considerable improvement in the patients’ condition. They held the perception that if the health workers were not competent, then there would not have been improvement in the patients’ condition.

*They are skillful and knowledgeable. The first thing is that there are positive changes in me. It’s not like my condition was bad but there were definitely some changes in me. Service user, depression*

Many service users and caregivers reported that the services they received from primary health care facilities were much better than the services at tertiary health facilities. Caregivers of patients with AUD also reported that health workers were doing their best and that because of their guidance and advice, the patients were now on the right path. On the contrary, a few caregivers thought that health workers did not have much information or experience. A patient with epilepsy mentioned that health workers did not show much concern for his problem or interest in his treatment, which led him to believe that health workers were not capable of treating epilepsy.

*My treatment could have been done in the primary health care facilities but the people of the primary health care facilities haven't shown much concern about this thing, so I haven't shown that much interest here.* ***Service user, epilepsy***

### Satisfaction with the services

Except for a few AUD cases, most service users and caregivers were satisfied with the services provided by primary health care workers. They cited free services and attentive health workers as major reason for satisfaction. One caregiver of a patient with psychosis said that her husband had thoroughly enjoyed the process of treatment, from getting medicine on a consistent basis to receiving psychosocial counseling. A number of service users and their caregivers spoke about health workers and staff from Transcultural Psychosocial Organization (TPO) Nepal paying home visits frequently and calling for updates on their condition. A caregiver (father) of a patient with depression reported:

*… TPO Nepal counselor used to come to my place frequently, tells me not to worry about my son, and provide love and care to him. He also told us that if people have such problems they can go to health post [i.e. primary health care facility] for treatment. He also requested us not to say bad things at them instead provide love and talk to them in a good way. Caregiver, depression*

Another factor contributing to satisfaction in cases of depression, epilepsy and psychosis was that patients’ condition improved after seeking services from the primary health care facilities and patients were also willing to take medicines as long as required. A caregiver of a patient with depression mentioned that the patient’s condition was improved after getting services from the primary health care clinic, although they had spent a sum of money in a private hospital. “*I had a good experience. I am happy as I am receiving lots of support from the health post for her. We have now been receiving the treatment as much as we have expected from them. I am satisfied with the service”.*

Some service users reported that primary health care workers also took advice from their seniors while treating their problem, which they considered positively. One participant with epilepsy shared her experiences saying that “*Overall, the experience was good since they provided me suggestions [counselling]. They also gave me medicines. Some health workers provided me suggestions by consulting with other doctors. They told me to take medicines for a certain time. They talked about reducing the dose of medicines after a certain time which was very good. I felt very good about the service. The service quality was good”.*

Psychosocial counselling service was considered valuable as patients and their families were able to share what they felt and thought about their problems with someone. Both service users and caregivers across all disorders reported that they benefited from the counselling service. Many of them believed that the counselling service was effective and more beneficial for improving their health condition and self-esteem. Participants with AUD spoke in many different ways about how health workers tried their best to help them quit drinking, which in turn helped to improve their relationships with their family, relatives and neighbors. One participant with AUD explained that counselling is needed in addition to medicines, particularly when the medicines do not work: “*I received both medication and psychosocial counseling; medication was the sample only but conversation [counseling] was the most effective. I felt happy when they talked to me. I reduced drinking alcohol substantially when they convinced me. Mostly the discussion part of the health post is better rather than the medication. The discussion part of health post helped me to gain knowledge and finally I was able to quit my drinking habit. Medication is like eating mud or flour but the knowledge is the best part”.- Service user, AUD.*

Other service users also reported that they felt better, happy, comfortable, and relaxed after getting to share their thoughts and problems with counsellors. Some reported that counseling had brought change in their symptoms, including staring blankly, wandering thoughts and suicidal thoughts. One of the patients with depression said that she felt relaxed by seeing the counselor and sharing all the things which she could not say otherwise. Another participant admired the counsellor for providing her adequate time: *“She [counselor] used to give me minimum 45 minutes at one visit, and the days when she could not come due to her small baby, she used to talk with me by telephone”.- Service user, depression.*

#### Barriers to receiving services

Despite a lot of positive feedback from both service users and caregivers about mental health services in primary care, some of them also reported various factors that created difficulties in initiating or continuing treatment. The most frequently reported barriers are discussed below.

### Medicine-related issues

Although the majority of the participants reported benefitting from the availability of the medicines free of cost, a few reported challenges in obtaining the required medicines free of cost from their primary care clinics. Some service users and caregivers reported that sometimes they had to purchase medicine from elsewhere because the medicines were frequently stock out in primary care clinics, which increased their expenditure on transportation and on medicine. Participants who had initiated treatment from other places and wanted to continue treatment with primary health care workers reported that the medicine at the primary health care facilities did not match the one they had received from other health institutions. Therefore, they stopped visiting primary health care facilities. A patient with psychosis described his experience, *I wanted a Nepali company’s medicine but they provided me an Indian one. That was the difference. If I had been provided the medicine that I take regularly then I would have come there.*

Availability of limited medicines in the primary health care facilities was another concern raised by the patients with psychosis and epilepsy.

*He [family member with psychosis] needed multiple medicines, however, only a few medicine was available in primary care. He didn’t get the complete doze of his medicines there, this was the reason we did not go for follow-up. If all the medicines are available in the health post then it would be good. -*

***Caregiver, Psychosis***

### Lack of a confidential space

Lack of a confidential place/room within health facilities for consultation and psychosocial counseling was reported as barrier for seeking services by many service users. Some of them reported that they felt uncomfortable sharing their problems in front of other people, and this was one of the factors leading to dissatisfaction with the services. For example, a patient with depression shared, “*In the health facilities…, they did not use to talk to me in a separate room. They used to talk to me in the common room where other patients are also treated. Sometimes nobody was while consulting whereas sometimes there used to be a few patients around”.* This was also supported by the response of another patient with depression. She reported, *“I used to talk more freely when there were a few people around; when there were [more] people around me, I used to talk much less”.*

### Different health workers at follow up visits

Unavailability of the same health worker at follow-up visits was another challenge reported by several service users, whereas some caregivers voiced their frustration over health workers not arriving at the health facilities on time. One of the patients with epilepsy reported, “*When I went there for the first time there was an old person [health worker] whereas there was a young person when I visited there second time. On another occasion, there was a senior doctor but later when I went there, there were young ones and again when I went later there were young female staff. So it’s hard to trust the treatment that they provide. It would have been good if I could meet with the same person again”.*

### Stigma and discrimination

Stigmatizing behavior of a few service providers, family and community members were reported as barriers for seeking mental health services by patients with all disorders. Caregivers also reported experiences of discriminatory behavior and negative comments from health workers because of their family member who had mental health problems. These experiences were reported frequently by caregivers of patients with AUD. A caregiver of a patient with AUD shared their experiences on how a service provider misbehaved to his/her relative having AUD, “*Doctors said, ‘why you have brought such drunkard person in the hospital, let him die’. They say many things to the people who drink alcohol. When we take him to the hospital for his serious problem they didn’t allow us to enter, they pushed us towards outside but anyhow we entered”.*

Caregivers also reported social stigma associated with mental illness in the community at large, explaining that some people suggested locking people with alcoholism up or restraining them with chains. The incidence of such social stigma forced caregivers to conceal the mental health condition of their family member, and some would go to the health facilities secretly to bring medicine for their family members in order to avoid people saying negative things about them. A caregiver of a patient with epilepsy described experiencing stigma:

*People don't like to come close to people with epilepsy in the time of falling down [during seizures] because [they think] this is a communicable disease and they have a fear that they will catch it****.***

On the other hand, they also reported that such stigmatizing behavior was not observed after improvement was seen among patients. One caregiver shared that after seeing the improvement in his/her family member’s condition, people in their neighborhood started referring others for treatment.

The most common types of stigma experienced by service users were negative stereotypes (e.g psycho, crazy), discrimination, social exclusion, and ill treatment. Most service users shared feeling excluded or devalued by their own family members and that they felt bad when their family members talked about them negatively with other people in the community and saw them as an embarrassment. Participants with psychosis reported frequent experiences of stigmatizing behavior because of their violent behavior. Some patients with psychosis also reported instances of human rights violation such as being tied or locked up in a room by their family members because their family members feared that they would harm other people.

### Poverty/low economic status

Most of the participants in the study were from a low economic status – such as farmers, those working on daily wages, or those working minimal pay jobs due to which they had difficulty managing basic expenses like food. Caregivers, in particular, shared instances of social compensation where they had cut down their food consumption to once per day to save money for the treatment of their family members. Caregivers were generally of view that their life has been affected by their family member’s mental health problems. A caregiver of a person with AUD reported that she had to earn herself in order to manage everything for her family because her husband lost his job due to his drinking habit

*“I have to feed my children, I have to educate them. Just think about it, a woman in the house whose husband’s condition is like that– she is working to feed her family, she has to look after her daughters who have grown up. Just imagine how much of a problem this is.”*

Caring for family members with mental health problem was experienced as a burden by a few caregivers because it created stress and placed an economic burden on the family. Interestingly, one caregiver described consumption of alcohol and poor economic condition as a vicious cycle of poverty: people drink mainly because of their financial problems, which arise in part due to problems in the family caused by the behavior of a family member with AUD.

#### Impact of the services

The most commonly reported impacts of the mental health treatment provided by trained primary care workers were improvement in individual health outcomes, able to involve in day-to-day activities, and improvement in household economic condition.

### Improvements in health outcomes

Improvements in symptoms severity were particularly reported among the patients with depression, psychosis and epilepsy. Caregivers of the patients with epilepsy as well as the patients with epilepsy reported that they stopped experiencing symptoms such as acting differently, foaming at the mouth, wandering aimlessly, dizziness, periodic seizures and falling unconscious after receiving treatment. One of the patients with epilepsy reported that she has not experienced any seizure since she started treatment from primary care. She said, “*Before starting medication, I used to have seizures 3 or 4 times to 8 or 9 times in a month but now that doesn’t happen. Now I am good. I have been carrying out my daily activities nicely. Now I don’t have a problem like before****.”***

Both service users and caregivers of people with psychosis and depression reported that they had observed a lot of improvement in their behavior. In comparison to their previous condition, they had less trouble falling asleep, less pain, and fewer suicidal thoughts, and they also felt an increase in appetite and self-esteem after the treatment. One caregiver of a patient with depression mentioned that the suicidal thoughts of her son had decreased substantially after receiving counselling from health workers and counsellors. “*If health workers and counsellors hadn’t looked after my son, then I don’t know what his condition would be like. He used to shout and cry, he used to carry rope and go towards our yard, he used to say different things [self-harm or suicide] and I used to convince him not to talk like that. They [health workers and counselors] were able to convince him. These days he doesn’t say anything like that”.*

In the case of AUD patients, none reported visible changes in their symptoms severity as like in other three disorders but reported feeling healthier and stronger than before. Increased appetite and weight gain after giving-up alcohol consumption were some of the notable improvements reported by alcoholic patients.

### Improvements in household economic condition

Availability of services free of cost was associated with improved economic condition of their family. Family members reported that service users were able to engage in income generating activities, which in turn supported the family economically. One of the caregivers of a psychosis patient reported – “*There have been changes in the economic condition of our family. After his health got improved, he now teaches. He also runs tuition classes for earning. Until the time there was improvement in his health, he used to take medicine and sleep. There wasn’t any income and it became very hard at that time in [regard to our] economic condition”.*

Improved condition of patients also led caregiver to spare time to engage in other economic activities which otherwise had been compromised earlier. “*She [patient] looks after the cattle. She prepares meal. She handles every little work of house. There is obviously some benefits sir, why wouldn’t there be. I also became able to walk outside in an independent way. I got a chance to handle my job. From this I could bring 2 or 4 thousand rupees and that would be useful for buying oil and spices.* ***Caregiver, psychosis.***

A patient of AUD shared he has been able to save Rs. 2–4000 now that he has given up drinking. Others also shared that they have saved transportation cost which otherwise would have been spent on visiting the health facilities.

### Involvement in day-to-day activities

Most of the caregivers reported that when their family members or relatives were having mental health problems, they had to spend much time taking care of them. After receiving the treatment from primary health care facilities, they were able to perform day-to-day activities such as taking care of themselves (e.g. maintaining hygiene, cleaning, washing clothes) cooking food, and doing household work; this allowed caregivers to become involved in other work. A caregiver of a patient with psychosis elaborated this saying: *“During the time when he doesn’t take his medicine, he doesn’t wake up even to wash his face. These days, he wakes up in time, bathes and does the work that he has. If there’s some work then he starts doing that in his own way. He does the work like building roofs for houses”.* This was also supported by the experiences of service users themselves, who described being able to do their personal work by themselves or help their family members with household chores. A patient with epilepsy explained how she started her day to day normal activities after treatment, “*I used to fall down while I would be cooking, doing farm work or doing any kind of house works. Now as I have realized, my problem has become much less. I do every work by myself as much as I could. Before I used to hire workers for every work but now I do everything as much as I could”.*

### Improvement in social life experiences

Participants also reported improved social life experiences and relationships after receiving treatment. Service users of AUD and their families were shunned and did not have good relations with the people in their communities. Family members of patients with AUD also reported being embarrassed and irritated by their drinking behavior. However, many service uses reported that the perceptions of community towards them had changed after the treatment. It was reported that their relationships were also improved with friends, family and relatives, and were also getting positive comments on their change. One caregiver of a patient with AUD reported that she had noticed a lot of changes in her brother after he gave-up drinking alcohol, specifically in his relationships with his children, wife and family. “*Yes a lot of changes in him, in the household activities, lots of changes in everything like change in the love for wife, children and in the family”.*

Similarly, another caregiver of a patient with psychosis reported similar experiences, where the family member with psychosis became more interactive than before the treatment. Some service users also reported that they were able to tolerate crowds and wanted to get involved in fun activities or in ceremonies. A patient with psychosis explained, *“Previously I used to sit alone, putting the hand on the forehead, holding the head by hand, playing wondering thoughts in heart, but now I like to get involved in fun activities”.*

## Discussion

To the best of our knowledge, this is the first study conducted among the people receiving mental health services in primary health care facilities and their caregivers in Nepal. People with a range of mental health problems, including depression, AUD, psychosis and epilepsy, were included in the study. Overall, both service users and their caregivers were positive and satisfied with the services provided by trained primary health care workers. The primary health care workers were provided mhGAP-IG based training in order to enhance their knowledge, skills and clinical competencies to provide basic psychological and pharmacological treatment [11]. Similarly, a separate cadre of psychosocial counsellors, deployed by the project, provided focused psychological interventions in the community [23]. Additionally, many service users had the opportunity to get services from mental health specialists during supervision (case conferences), which may have contributed to the high level of satisfaction among service users and caregivers. Most importantly, availability of both psychological and pharmacological services free of cost in their own community was highly appreciated because this helped to save travel time as well as treatment and travel costs.

Many participants regarded psycho-education and psychosocial counseling as a practice of giving advice or suggestions; it was generally considered to be more effective and useful than pharmacological treatment. The possible reasons for preferring psychological treatment could be the involvement of family members in the counselling sessions, home visits by the community counsellors – which were less stigmatizing and more confidential, and the use of less stigmatizing language, with counsellors using terms such as ‘heart-mind problems’ or focusing on the symptoms reported by patients rather than using the word ‘mental health’ in the counseling sessions. After the treatment, significant improvements were reported in patients’ health conditions and outcomes; subsequently many participants were found to be engaged in their regular day-to-day activities such as taking care of the domestic animals, involvement in farm-related activities and other income generating activities. These results are also supported by our previous studies where primary care-based mental health treatments were effective in improving individual-level functioning and clinical outcomes [15, 24]. These results are also consistent with the findings of a trial in India where trained lay-counselors delivering primary care-based mental health treatment were effective in improving recovery from depression and anxiety [25]. A recent study in eastern Nepal also reported that the trained primary health care workers successfully provided psycho-education and counselling services at primary care [26].

Despite the positive attitude of service users and caregivers towards primary care-based mental health services, they also reported various barriers and challenges to receiving mental health services in primary care. Since only one medicine for each disorder was made available by the PRIME study, a few service users and caregivers - particularly those requiring multiple medicines – expressed dissatisfaction with the services. Similarly, participants frequently mentioned unavailability of psychotropic medicines in the health facilities, which could be due to the lengthy administrative process for procurement and distribution of medicines resulting in the frequent stock-out of medicines. In addition, due to a blockade in the Indo-Nepal border in 2015, there was scarcity of fuel, which created problems for the supply of medicines to the respective health facilities for several months. Problems in regular supply of psychotropic medicines have also been reported in various studies, and this is considered as one of the biggest challenges to integration of mental health services into primary health care system [27]. Frequent transfer of trained health workers was also identified as an area of dissatisfaction among service users and caregivers. They reported that they were not able to meet or consult with the same health worker in their follow-ups, which led to the loss of trust towards services.

Lack of confidentiality in the health facilities and short consultation time with health workers was also reported as one of the causes of dissatisfaction among service users. Shortages of human resources and lack of appropriate infrastructure have also been reported as key barriers to accessing mental health services in primary care in South Sudan [28], Uganda [29] and six LMICs [30].

Another challenge and source of dissatisfaction reported by service users was the stigmatizing behavior of service providers, community members and their own family members. Negative attitudes among primary care workers were particularly reported by patients with AUD and their caregivers. Stigmatizing and negative attitude of service providers toward people with mental illness have been reported in various other studies. For example, widespread stigma associated with primary health care workers who were supposed to act as frontline staff in the delivery of mental health care was reported as one of the primary obstacles for integration of mental health services in primary care in Zambia [31]. Similar results have been reported by a study conducted with a wide range of policy makers in six countries from Asia and Africa (i.e. Uganda, South Africa, Nigeria, Nepal, India and Ethiopia), where negative attitudes of service providers towards people with mental illness was identified as an important supply-side barrier to seeking mental health care across all countries [30]. Similarly, a study conducted with medical professionals (faculty members and postgraduate trainees) in Southern India reported that a significant number of the medical professionals had negative attitudes towards people with mental illness [32].

The findings of this study may have several implications for improving access and quality of mental health services in primary and community health care settings in Nepal. First, both service users and caregivers reported that psychological interventions, particularly the psychosocial counseling service, were more effective than pharmacological treatment. In PRIME, a separate cadre of psychosocial workers (i.e. community counsellors) was recruited to provide counselling services in both health facilities and community settings. The findings of this study have also been supported by our previous randomized controlled trial in which people receiving both health facilities-based services and community counseling had better health outcomes compared to those who received health facility-based services only [33]. Therefore, a separate cadre of psychosocial workers is recommended for providing psychological treatments in primary health care and community settings.

Second, due to the lack of confidential places for consultation, many service users reported that they felt reluctant to share their problems with the health workers in front of many people. In addition to this, perceived stigma and discrimination was also reported as one of the major reasons for not receiving treatment for depression and AUD in Chitwan [34]. Similarly, lack of confidential space has also been reported as one of the most important system-level barriers to integrating mental health services into primary care by the trained health workers [35]. Therefore, a separate and confidential place for consultation and psychological intervention should be made available in each of the health facilities.

Third, unavailability of required medicines in health facilities and frequent stock-out of the available medicines have been reported as reasons for service users dropping out of treatment. There could be adverse consequences in patients’ treatment and recovery processes if psychotropic medicines are out of stock for long periods of time. In the existing health care system, limited psychotropic medicines are made available in primary care, and the patients who require multiple medicines have to manage medicines themselves. Therefore, the current drugs procurement process needs to be revised to facilitate quick and efficient supply of medicines, and a range of psychotropic medicines should be added in primary care.

Fourth, stigmatizing attitudes and misbehavior of service providers towards people with mental illness have been reported by a number of service users and caregivers, especially by patients with AUD. Previous studies have also shown that the negative attitude of health workers is considered a barrier to utilizing health care services [36]. Therefore, an anti-stigma program should be developed and implemented to minimize mental health stigma associated with service providers.

Fifth, frequent transfer of trained health workers or unavailability of the same health workers in follow-up visits was reported as an important barrier to continuation of mental health services in primary care. Frequent transfers may reduce motivation of health workers, and have negative implications for service delivery. Therefore, transfer of health workers should be regulated, and a standard protocol should be developed for transferring health workers from one health facility to another. In PRIME we trained all primary health care workers on mental health. This system allowed every health worker to be involved in mental health service delivery; however, provision of a dedicated mental health worker could be one of the strategies to minimize these barriers. This strategy might also be helpful to minimize stigma associated with mental illness.

Finally, many caregivers reported that due to their continuous involvement in care and support of their mentally ill family members, they also felt stressed and anxious. The persistent stress of caregiving may also adversely affect caregivers’ physical and mental health well-being, which consequently may produce burnout and emotional exhaustion. Therefore, a community-based psychological intervention needs to be developed and implemented to support family members of people with mental illness.

This study has some limitations. First, the study was conducted in Chitwan, a relatively accessible district in terms of geography in Nepal. The study VDCs are well connected with roads and other modern facilities compared to other VDCs within the district. Therefore, the results of the study may not be generalized in other districts where health facilities are scattered in more remote areas. Second, the study was conducted with participants who were involved in the PRIME cohort study, and they had already been contacted multiple times by the research team. This might have influenced service users and caregivers to provide socially desirable responses. Finally, we applied a purposive sampling method in the study so the results may not be representative of the entire population.

## Conclusion

This study examined the perspectives of service users and caregivers on primary care-based mental health services, including their perceptions on the accessibility, acceptability, and impact of the services as well as barriers to the initiation and continuation of treatment. The results suggest that both service users and caregivers were positive and satisfied with the mental health services available in the primary and community care setting. They also perceived health workers to be competent and skillful in providing mental health services. Although, both psychological and pharmacological services were made available free of cost in the health facilities and community, participants considered psychological services more effective than pharmacological treatment. The major barriers that hindered the utilization of primary care-based mental health services included frequent transfer of trained health workers, different health care providers at follow-ups, frequent stock-out of medicines or unavailability of the required medicines, lack of a confidential place in the health facilities for consultation, and stigmatizing and negative attitudes of some of the health care providers towards patients. The key changes that need to be implemented in order to improve access and quality of primary care-based mental health services included the provision of a separate cadre of psychosocial workers to provide psychological interventions, quick and efficient mechanisms for procurement and supply of psychotropic medicines, provision of confidential places in health facilities, and further training of health workers to minimize stigma and negative attitudes associated with mental illness.

## Data Availability

Interested parties may notify the PRIME investigators of their interest in collaboration, including access to the data set analyzed here, through the following website: http://www.prime.uct.ac.za/contact-prime.

## Abbreviations

AUD: Alcohol use disorder
LMIC: Low and middle income countries
MHCP: Mental health care plan
MNS: Mental, neurological and substance use disorder
PHC: Primary health care
PRIME: Programme for improving mental health care
VDC: Village development committee
WHO: World health organization
